# Self Medication

**Published:** 1859-01

**Authors:** 


### SELF MEDICATION.

Of any four persons met successively on the street, three will strongly inveigh against taking medicine and against the doctors, and multitudes of publications are scattered through the land every day by a class of persons as reckless and impudent as they are ignorant, assuming to themselves the name of "reformers," their papers being the vehicles of their trumpery, making all sorts of imaginary and impossible statements as to the ravages of what they call "druggery," and fighting under the popular banner of "temperance," with maudlin professions about "progress," "human amelioration," "elevation of the masses," "equality," "fraternity," and all that, and last, but not least, pandering to the passions of a depraved nature, they stab secretly, and behind, and under cover of false garbs, the fundamental principles of our holy religion, and indeed of all religion, and by these means have got up such a hue and cry against physic, that even medical men, despicably weak-minded of course, take up the refrain, chime in with the prejudices of a gullible community, and are getting into the way of prescribing almost no medicine at all, in cases where it was urgently demanded, doing violence to their own better judgment, rather than incur the hazard of censure, in case the disease should take a fatal turn. On the other hand, as among the people themselves there is a most extraordinary paradox, in that they have fallen into the habit of swallowing medicine on their own responsibility, or by the advice of any ignoramus or knave who may happen to fall in with them, and this too for ailments so trifling sometimes, that simple rest and warmth for a few hours would restore them to usual health.

Not long ago a lady near us gave a little girl a dose of castor oil for what appeared to her to be a little cold. This acted on the bowels freely, and, by weakening the system, took from it the power of throwing out the real disease on the surface, and the only child of wealthy parents died in forty-eight hours of undeveloped scarlet fever.

More recently, a man felt unwell, and concluded to cure himself by mixing with a pint of beer a tablespoonful of salt, a raw onion, and twenty-five cents worth of quinine. Soon after taking it, vomiting set in, and he died in twenty-four hours. Fools cannot die off too soon; but we earnestly advise all whose lives are of worth in the community in which they live that in any case where, in their own opinion, they are ill enough to require medicine, swallow not an atom by any body's advice, however simple the remedy may appear, but send at once for a respectable physician. The remedy advised may do no harm, if it does no good; but even in that event, it may cause a loss of time in waiting for its effects which no medical skill may be able to make up for.

